# Analysis-ready satellite data mosaics from Landsat and Sentinel-2 imagery

**DOI:** 10.1016/j.mex.2022.101995

**Published:** 2023-01-04

**Authors:** Hans Ole Ørka, Jãnis Gailis, Mathias Vege, Terje Gobakken, Kenneth Hauglund

**Affiliations:** a Faculty of Environmental Sciences and Natural Resource Management, Norwegian University of Life Sciences, P.O. Box 5003, Ås NO-1432, Norway; b Science [&] Technology Corporation, MESH, Tordenskioldsgate 6, Oslo NO-0160, Norway

**Keywords:** Earth observation, Remote sensing, Optical satellite imagery, Preprocessing, Land cover classification, Geomosaic - analysis-ready satellite data mosaics

## Abstract

Today's enormous amounts of freely available high-resolution satellite imagery provide the demand for effective preprocessing methods. One such preprocessing method needed in many applications utilizing optical satellite imagery from the Landsat and Sentinel-2 archives is mosaicking. Merging hundreds of single scenes into a single satellite data mosaic before conducting analysis such as land cover classification, change detection, or modelling is often a prerequisite. Maintaining the original data structure and preserving metadata for further modelling or classification would be advantageous for many applications. Furthermore, in other applications, e.g., connected to land cover classification creating the mosaic for a specific period matching the phenological state of the phenomena in nature would be beneficial. In addition, supporting in-house and computing centers not directly connected to a specific cloud provider could be a requirement for some institutions or companies. In the current work, we present a method called Geomosaic that meets these criteria and produces analysis-ready satellite data mosaics from Landsat and Sentinel-2 imagery.

• The method described produces analysis-ready satellite data mosaics.
• The satellite data mosaics contain pixel metadata usable for further analysis.
• The algorithm is available as an open-source tool coded in Python and can be used on multiple platforms.

## Graphical abstract

**Figure:**
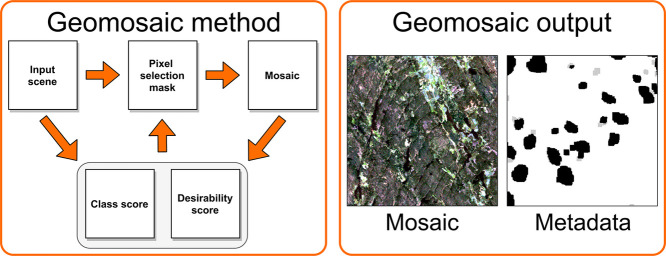

Specifications table

**Table:** 

Subject Area:	Agricultural and Biological Sciences
More specific subject area:	Remote sensing
Method name:	Geomosaic - analysis-ready satellite data mosaics
Name and reference of original method:	European Space Agency (2021). *Sen2Three*. STEP - Scientific Toolbox Exploitation Platform. (http://step.esa.int/main/snap-supported-plugins/sen2three/). Accessed on 18 Jun. 2021. Griffiths P., van der Linden S., Kuemmerle T., Hostert P. (2013). A Pixel-Based Landsat Compositing Algorithm for Large Area Land Cover Mapping, IEEE Journal of Selected Topics in Applied Earth Observations and Remote Sensing. 6:2088–2101. https://doi.org/10.1109/JSTARS.2012.2228167. Hagolle O., Morin D., Kadiri M. (2018). Detailed Processing Model for the Weighted Average Synthesis Processor (WASP) for Sentinel-2. http://dx.doi.org/10.5281/zenodo.1401360. Kempeneers P., Soille P. (2019). Mosaic of Copernicus Sentinel -2 data at global scale. European Commission, JRC116553. Li H., Wan W., Fang Y., Zhu S., Chen X., Liu B., Hong Y. (2019). A Google Earth Engine-enabled software for efficiently generating high-quality user-ready Landsat mosaic images. Environmental Modelling & Software 112: 16–22. https://doi.org/10.1016/j.envsoft.2018.11.004. World Bank. (2020). Satellite Monitoring for Forest Management: Use of Earth Observation Tools in the Monitoring of Tropical Dry Forests. World Bank, http://hdl.handle.net/10986/34998. Zobrist A.L., Bryant N.A., McLeod R.G. (1983). Technology for large digital mosaics of Landsat data. Photogrammetric Engineering and Remote Sensing 49: 1325–1335.
Resource availability:	https://gitlab.com/stcorp-public/geomosaic

## Method details

Methods for combining multiple single scene satellite imagery have been used for many decades [1]. The, early methods consist of merging a few selected scenes [1]. However, multiple scenes are used as more high-resolution satellite imagery has become freely available, e.g., from the Landsat archive [2] and as imagery from the Copernicus Sentinel-2 satellites [3]. As a result, many mosaics e.g., [4,5] and tools to create such exists e.g. [6], [7], [8], [9], [10]. Mosaicking refers to combining two or more spatially independent images into one continuous image, while compositing refers to combining multiple overlapping images into one using an aggregation function [11]. The current method focuses on combining several independent images without any aggregation function; thus, we refer to the method as mosaicking. Some of the existing mosaics are only static and application-specific tuning is impossible [4,5]. Other methods may not provide pixel-level metadata that can be used in modeling, classification or stratification [8,10]. For example, when creating data mosaics based on aggregating pixel values from the same locations e.g., averaging methods such metadata are not available [8]. However, such mosaics may be appropriate for certain applications, but in other applications metadata may be desired. Furthermore, many of the mentioned mosaicking tools only work on a specific type of satellite data as Landsat 8 or Sentinel-2 data [6]. At last, mosaicking tools could be proprietary or connected to a specific software or cloud platform [9]. Based on these issues, we established a generic mosaicking method working on both Sentinel-2 and Landsat 4-8. Thus, we developed the Geomosaic method that implements a configurable smart pixel selection method that lets the user adjust the pixel selection criteria to fit the particular use case. The Geomosaic method is implemented in a software tool developed in Python and is available at Gitlab under a MIT license.

The Geomosaic tool has specific implementations for: (1) Sentinel-2 surface reflectance data (L2A), (2) Landsat 8 Collection 1 Surface Reflectance data (LaSRC - OLI/TIRS) and (3) Landsat 4-7 Collection 1 Surface Reflectance data (LEDAPS - TM/ETM+). Spatiotemporal data aggregation is performed by (1) sorting the input scenes by date, (2) reprojecting the scene to the desired target projection if needed, (3) calculating the spatial extents of potential contribution from the scene, and (4) generating a pixel selection mask at each step. The pixel selection mask is established using the Geomosaic method described below.

**Fig. 1:**
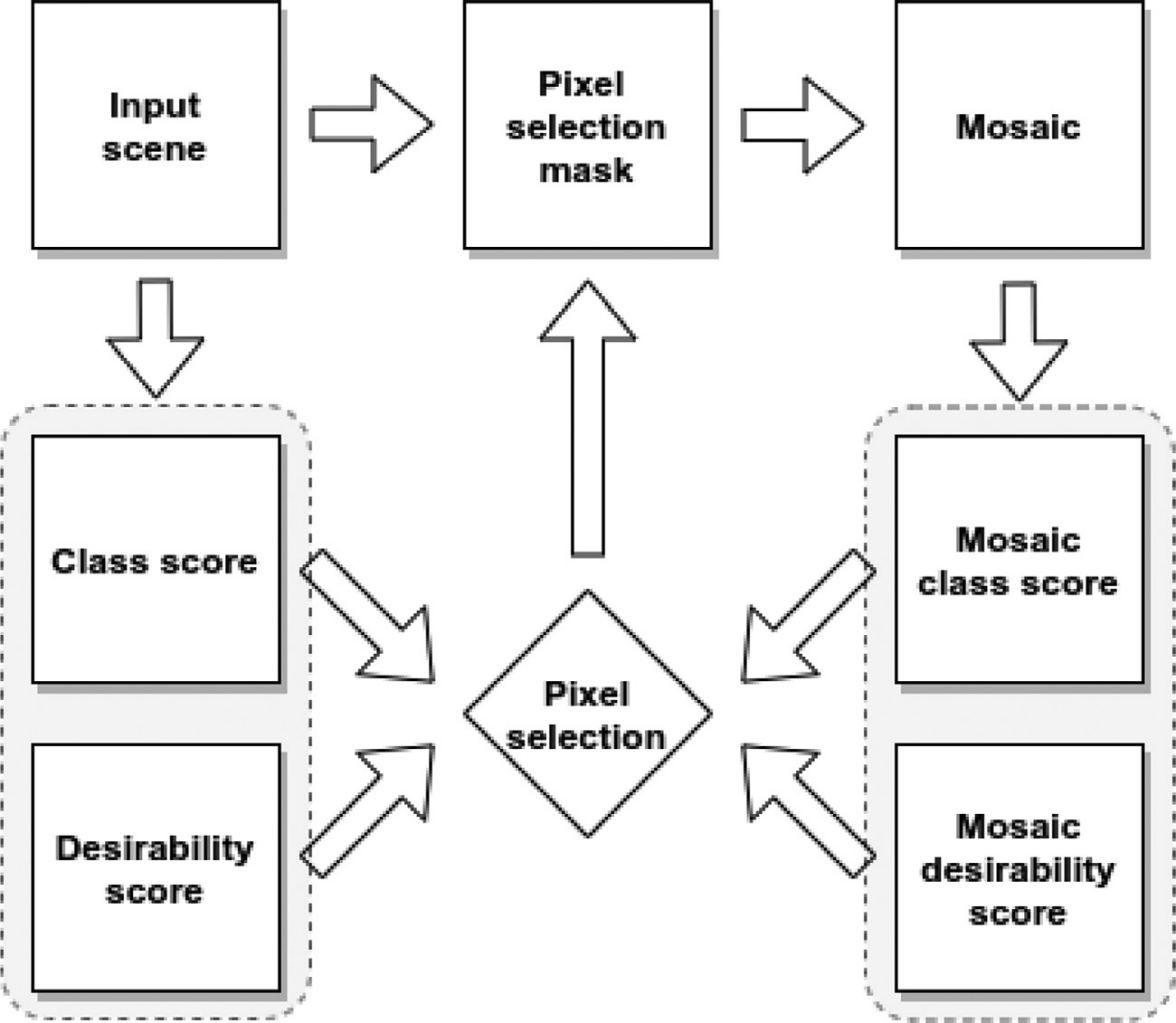
Schematic overview of the Geomosaic method.

The Geomosaic method uses a two-step pixel selection procedure based on a class score and a desirability score. Thus, for each pixel in the mosaic pixels are updated based on the pixel selection mask created from these two score values. If the new scenes’ pixel has a larger class score or the same class score, but a larger desirability score the pixel values for all bands in the mosaic are updated (Fig. 1).

In the first step in the Geomosaic method, the class score is established by selecting pixels according to a class categorization, where pixels from ‘worse’ classes are progressively exchanged for ‘better’ classes, according to a class hierarchy (Table 1). The classes used in the hierarchy for Sentinel-2 was adapted from the Sentinel 2 MSI Technical Guide [12]. For Landsat 4-8 the classes described in the Land Surface Reflectance Code Product Guide V3.0 [13] was used. The class hierarchy and respective class scores used was fixed in the Geomosaic tool (Table 1). Nevertheless, other classes and another hierarchy of the classes can be used with the Geomosaic method.

**Table 1:** Class hierarchy adopted to compute class score and pixel quality. A larger class score means a better pixel quality, i.e., a score of 1 is the worse pixels while a score of six or seven is the best quality for Sentinel-2 or Landsat, respectively.

Class score	Sentinel –2	Landsat
1	no_data, saturated, defective	no_data, saturated
2	cloud_medium_probability, cloud_high_probability	terrain_occlusion
3	thin cirrus	cloud_medium_probability, cloud_high_probability
4	snow/ice	cirrus_medium_probability, cirrus_high_probability
5	Dark, shadow, unclassified	snow/ice
6	vegetation, soil, water	dark, shadow
7		clear_terrain, water

In the second step, a weighted pixel desirability score is calculated for each scene:

$$S_{des}=w_{date}S_{date}+w_{gpix}S_{gpix}+w_{aot}S_{aot},$$

where, $S_{date}$ describes the relative distance from the desired mosaic target date, $S_{gpix}$ is a ratio of good pixels in the scene against all pixels and $S_{aot}$describes the mean aerosol optical thickness of the scene, and $w_{date},w_{gpix},w_{aot}$ are relative weights that are summarized to one:

$$w_{date}\in \left[0,1\right],$$

$$w_{gpix}\in \left[0,1\right],$$

$$w_{aot}\in \left[0,1\right],$$

$$w_{date}+w_{gpix}+w_{aot}=1,$$

These weights can be changed in order to adapt the mosaic to a specific problem or application, e.g., by increasing the value of $w_{date}$ to have input images closer to the target date or increase the value of $w_{gpix}$ to have a higher spatial homogeneity of the mosaic or alternatively increase the atmospheric clarity of the mosaic by increasing $w_{aot}$.

**Fig. 2:**
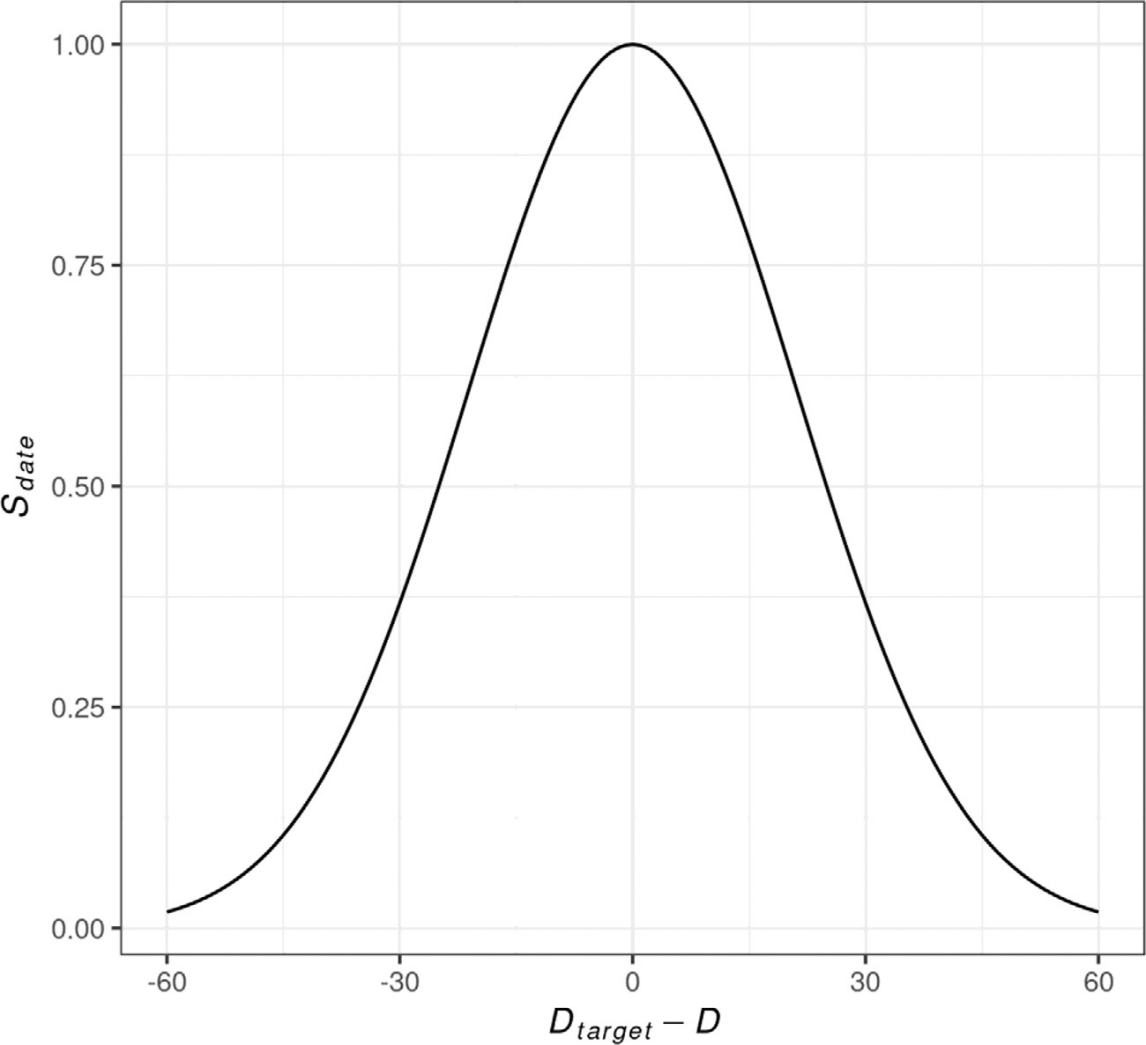
The value of $S_{\text{date}}$ based on the difference between the target date and the date of the input scene (σ^2^ = 30).

The variable $S_{date}$ is a value between 0 and 1 in a quasi-Gaussian distribution:

$$S_{date}=exp\left(-{\left(\frac{D_{target}-D}{\sigma^{2}}\right)}^{2}\right),$$

where, *D_target_* describes the target date for the mosaic, *D* describes the date of the current scene and *σ^2^ is* the standard deviation in days. The value used for *σ^2^* in the Geomosaic tool was 30 days. The value of $S_{date}$ is one if the image is from the desired date and will decrease as the difference between the target date and the scene date increases. Images from +/- 30 days are strongly preferred over other dates due to the selected standard deviation (Fig. 2).

The ratio of good pixels in the scene against all pixels $S_{gpix}$is computed as:

$$S_{gpix}=\frac{\sum_{i=1}^{n}gpix_{i}}{n},$$

where, *n* is the total number of pixels in the scene and $gpix_{i}$takes the value 1 if the pixel *i* is classified as good and 0 if not classified as good. The pixel is classified as good if the pixel has the highest class score possible for the given satellite data type and typically denoting a cloud, snow, and artefact free pixel (i.e., class score 6 for Sentinel-2 and class score 7 for Landsat, see Table 1).

The mean aerosol optical thickness of the scene is described with $S_{aot}$ and calculated as follows:

$$S_{aot}=1-\frac{\sum_{i=1}^{n}aot_{i}}{n},$$

where, $aot_{i}$ is the “aerosol optical thickness” of pixel *i*, and *n* is the number of pixels.

When performing the spatiotemporal data aggregation of the sorted input scenes, all pixels in the mosaic with a smaller class score than the potential new scene are replaced. Furthermore, for pixels who have the highest possible class score in both the input scene and the mosaic, the pixel is exchanged only if the desirability score for the input scene *S_des_* is higher than the *S_des_* of the pixel currently in the mosaic. In addition to the mosaic, the algorithms create a tilemap that contains a metadata identifier to the original scene. In the Geomosaic tool provided, this value is stored as an unsigned integer, and the metadata is stored in an output GeoTiff file. Thus, each pixel in the mosaic is easily traceable back to the original input scene from which it was selected.

**Fig. 3:**
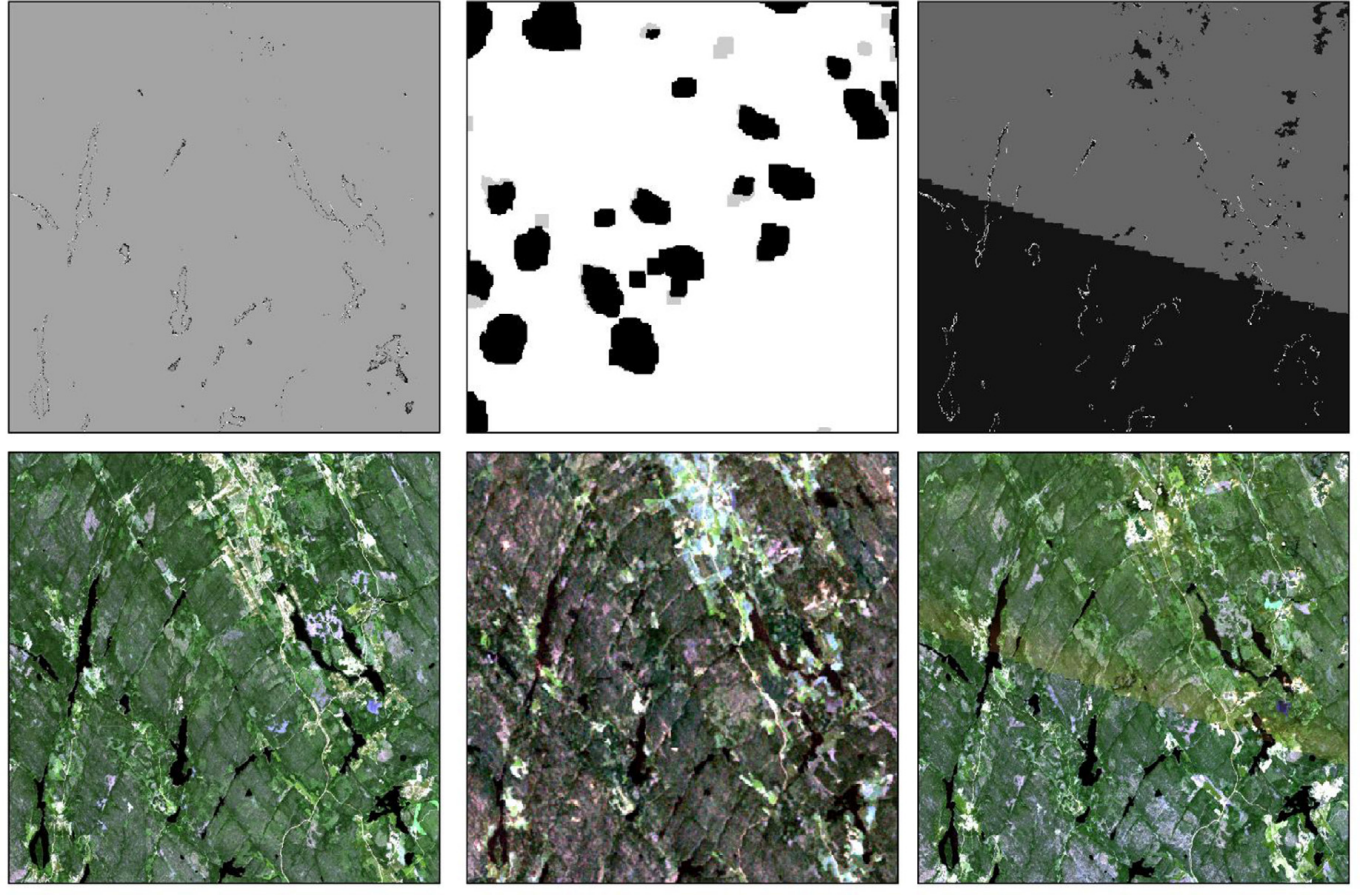
Example of tile maps (top) and mosaics (bottom) from different types of satellites (from left Sentinel-2 from year 2020, Landsat from year 1986, and Sentinel-2 from 2018).

The Geomosaic method is applicable to several use cases and types of satellites data and provides a mosaicking method that enables user-specific inputs and provides analysis-ready satellite data mosaics. See examples of tile maps and mosaics in Fig. 3.

## Declaration of Competing Interest

The authors declare that they have no known competing financial interests or personal relationships that could have appeared to influence the work reported in this paper.

## Data Availability

No data was used for the research described in the article.
